# A pilot metabolomic study of drug interaction with the immune response to seasonal influenza vaccination

**DOI:** 10.1038/s41541-023-00682-2

**Published:** 2023-06-12

**Authors:** Amnah Siddiqa, Yating Wang, Maheshwor Thapa, Dominique E. Martin, Andreia N. Cadar, Jenna M. Bartley, Shuzhao Li

**Affiliations:** 1 The Jackson Laboratory for Genomic Medicine, 10 Discovery Drive, Farmington, CT 06032 USA; 2 Department of Immunology and Center on Aging, University of Connecticut School of Medicine, 263 Farmington Avenue, Farmington, CT 06030 USA

**Keywords:** Biotechnology, Medical research

## Abstract

Many human diseases, including metabolic diseases, are intertwined with the immune system. The understanding of how the human immune system interacts with pharmaceutical drugs is still limited, and epidemiological studies only start to emerge. As the metabolomics technology matures, both drug metabolites and biological responses can be measured in the same global profiling data. Therefore, a new opportunity presents itself to study the interactions between pharmaceutical drugs and immune system in the high-resolution mass spectrometry data. We report here a double-blinded pilot study of seasonal influenza vaccination, where half of the participants received daily metformin administration. Global metabolomics was measured in the plasma samples at six timepoints. Metformin signatures were successfully identified in the metabolomics data. Statistically significant metabolite features were found both for the vaccination effect and for the drug-vaccine interactions. This study demonstrates the concept of using metabolomics to investigate drug interaction with the immune response in human samples directly at molecular levels.

## Introduction

The human immune system is our defense against infectious agents and malignancy, but also susceptible to many signaling cues^1–6^. The dysfunction of immune system often underlies a large number of autoimmune, metabolic and inflammatory conditions^1,7–9^. The health burden is significantly greater in the ageing population^10^, which commonly live under pharmaceutical medications, and respond poorly to many vaccines^11^. It is clearly important to understand how pharmaceutical drugs interact with the immune system, but it is not an easy task given that human immunology is still a rapidly evolving science^12,13^. In recent years, much progress in human immunology was achieved via vaccine studies, as vaccines are an excellent tool to probe human immune system providing an opportunity to learn about immunological molecular perturbations over time from days to years^14^.

Limited epidemiological studies have reported evidence of small molecular drugs impacting human immune responses, e.g., administration of statins has a minor detrimental but significant effect on influenza vaccination^15–17^. Using hospital records, Kidd et al. showed that a number of small molecular drugs influence the numbers of white blood cell subsets^18^. These hospital records are not easy to match to immunological studies, and they are not meant to be comprehensive. Furthermore, direct measurement of concentration of small molecular drugs and their metabolites, similar to the tests on professional athletes for prohibited drugs, is the most important information. Because it reflects the ongoing biological state and varying metabolic rate among individuals. In this regard, the advancement of mass spectrometry based metabolomics is potentially a game changer.

Metabolomics is the global measurement of small molecules in a biological system, which includes biological metabolites, dietary intake, microbial contributions, environmental pollutants, and often pharmaceutical drugs^19–22^. Liu et al. recently showed successful identification of nicotine (tobacco), naphthol sulfate (industrial chemical), omeprazole (medication) and piperine (food) and their derivatives in routine metabolomics analysis of human samples^23^. With both the environmental factors and biological responses in the same data, a new paradigm of gene-metabolome-environment interaction is emerging^24,25^. Thus, in a controlled study of vaccine induced immune responses, metabolomics offers the opportunity to examine both the drug response and vaccine response, and test for potential interactions.

The application of metabolomics to vaccinology is still in a nascent stage. Previous metabolomic analyses showed that systemic metabolites were perturbed by seasonal influenza vaccine^26^ and by herpes zoster vaccine^27^, with broad interactions with cellular and gene programs. A significant observation in the integrative analysis of human transcriptome and metabolome in Li et al. (2017)^27^ was that baseline SREBF1 (Sterol regulatory element-binding protein 1) activity was associated with B cell responses to vaccination, and the finding was recently confirmed in a detailed mouse study^28^.

Here, we have conducted a pilot study of seasonal inactivated influenza vaccine (IIV) in older adults, where half of the participants received metformin, a common drug for controlling diabetes, for a total of 20 weeks. The study was double-blinded and placebo-controlled, enrolling nondiabetic/nonprediabetic men and women over the age of 65 years. Ultrahigh-resolution metabolomics was performed on the plasma samples from 15 participants over six timepoints. The study was motivated by the effects of metformin in improving mitochondrial functions, reducing chronic pro-inflammatory signaling^29^, and targeting multiple hallmarks of aging^30^. Given the prominent role of metformin in aging studies and its geroprotective potential at molecular level^31^, it becomes an important question if metformin enhances the immune responses to vaccines. Research in this direction is urgent because better vaccine efficacy is needed for the older population and how to optimize their immunity has broad health impacts. In this report, we focus on how the metabolomics data manifest the biological responses to metformin, IIV vaccine and their interaction.

## Results

**Fig. 1:**
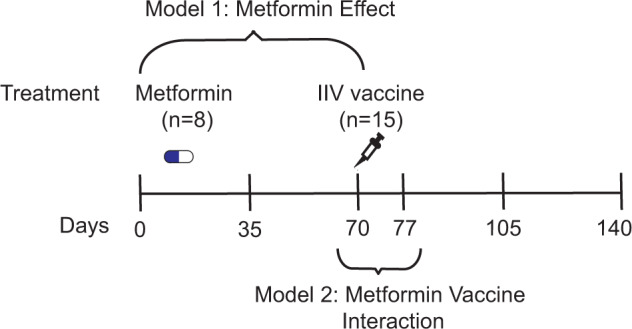
A double blinded clinical study of metformin in influenza vaccination in the elderly. A total of 15 study participants over the age of 65 years were randomly assigned to metformin or placebo treatment for 20 weeks. All participants were vaccinated with high-dose trivalent inactivated influenza vaccine after 10 weeks of treatment of metformin or placebo. Blood samples were collected over six timepoints, on days 0, 35, 70, 77, 105 and 140 approximately. Metformin administration started on day 0, and vaccine was administered on day 70. Our statistical analysis used two models to focus on the metformin effect (Model 1 using first three timepoints), and on the vaccine effect and interaction (Model 2 using two timepoints before and after vaccination).

Fifteen volunteers were recruited at the University of Connecticut Health Center, and randomly double-blinded assigned into two groups for either placebo (*n* = 7) or metformin (*n* = 8) treatment. The cohort was between 67 to 89 years old, with 8 males and 7 females and no significant differences in basic characteristics at baseline (placebo: 74.71 ± 2.45 years old, 3 males, BMI: 27.31 ± 1.68; metformin: 74.13 ± 2.42 years old, 5 males, BMI: 26.43 ± 1.47). The metformin group received Metformin Hydrochloride Extended-Release, 1500 mg/day (three 500 mg ER tablets once a day, starting at 500 mg ER/day and progressed per current recommendations). High dose trivalent inactivated influenza vaccine (Fluzone, Sanofi Pasteur Inc) was administered via intramuscular injection to all participants at approximately day 70 (Fig. 1).

The antibody and T cell responses in this cohort are published elsewhere^32^. Briefly, similar increase of antibody titers was observed post vaccination in both the metformin and control groups. Decreased CD57 expression was observed in CD4 T cells but not in CD8 T cells. Overall, the immunological data showed some trending improvements with metformin for flu vaccine responses, including circulating T follicular helper cells, but the cohort was underpowered for full conclusions. However, the adaptive responses are only part of our highly complex immune system. Therefore, in order to gain in-depth molecular insights, we analyzed the metabolomic profiles in this cohort.

### Untargeted metabolomics measured metformin and its abundance in the plasma of study participants

The plasma samples collected from the participants were analyzed by ultrahigh-resolution metabolomics. We applied four untargeted LC-MS (liquid chromatography-mass spectrometry) methods to increase the coverage of assays: hydrophilic interaction chromatography (HILIC) with positive electrospray ionization (ESI + ) and with negative electrospray ionization (ESI-), reversed phase (RP) chromatography with ESI+ and ESI−. The numbers of metabolite features in each method are reported in Table 1, after filtering of background peaks and by signal-to-noise ratio (SNR).

**Table 1:** Summary of significant metabolite features in statistical models.

		HILIC ESI+	HILIC ESI−	RP ESI+	RP ESI−
Total # Features		5987	4245	3284	8546
Model1	Drug Response	58	179	83	176
Model2	Vaccine Response	19	07	46	46
	Drug Response	06	02	03	02
	Drug Response * Vaccine Response	02	0	0	01

Significance is defined by FDR < 5% and absolute fold change response >1.5 in both post-metformin visits compared to baseline in Model 1 or after and before vaccination in Model 2. See Method for detail of the statistical models.

**Fig. 2:**
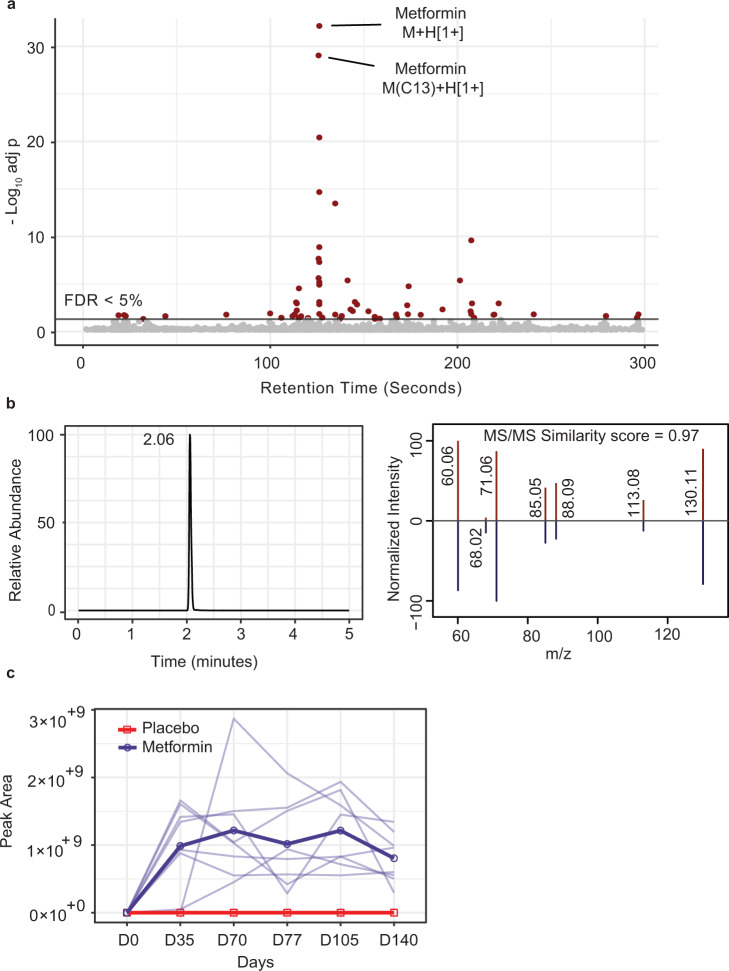
Identification of metformin and measured kinetics in study cohort. **a** Metabolite features that are different between the metformin group and placebo group, analyzed using Model 1, a mixed effect model where visit was modeled as fixed effect and participants were modeled as random effect. Significance is shown as -log_10_(adjusted *p*-value) on Y-axis. The two most significant features correspond to metformin and its ^13^C isotopologue. Features with false discovery rate (FDR) under 0.05 are colored in red. **b** Metformin is identified by accurate mass and fragmentation in MS/MS. Reference MS/MS spectrum of metformin is from MassBank (id: EA255011; red color), precursor ion m/z 130.1089. **c** Kinetics of metformin in all study participants. No metformin is detected in the placebo group (red). Each participant in the metformin group is plotted in light blue, and their mean values are in dark blue. All data in this figure are based on ESI+ mass spectrometry coupled with a HILIC column.

As a first step, the global metabolomics data enabled us to investigate the metabolic impact by metformin administration. This was analyzed by the time course in the metformin treated participants, using the three timepoints prior to vaccination (days 0, 35 and 70 as depicted in Fig. 1). Since metabolomics not only measures biological metabolites but small molecules in general, it is not surprising to find metformin itself in the metabolomics data. Indeed, the most significant two features were metformin and its ^13^C isotopologue (Fig. 2a). The isotopologue was from the naturally occurring stable ^13^C carbon atoms and eluted at the same time as the more abundant ^12^C form in chromatography. The LC-MS spectra of metformin and its identification via MS/MS are shown in Fig. 2b. The feature intensity values (peak area in LC-MS) in metabolomics are a proxy of the concentration in biological samples. With metformin identified, its abundance in the study participants was plotted in Fig. 2c, where the metformin group show a persistent level of metformin through the course of this study and the placebo group have no detected level. Individual variation is also seen in Fig. 2c, which reflects the heterogenicity of human populations, including the different metabolic rates among individuals. It cannot be ruled out that individuals might have different compliance to the study regimen. These data prove that valuable pharmacological information can be directly obtained from metabolomics without clinical records.

### Metformin induced broad metabolomic changes, including fatty acid biosynthesis

**Fig. 3:**
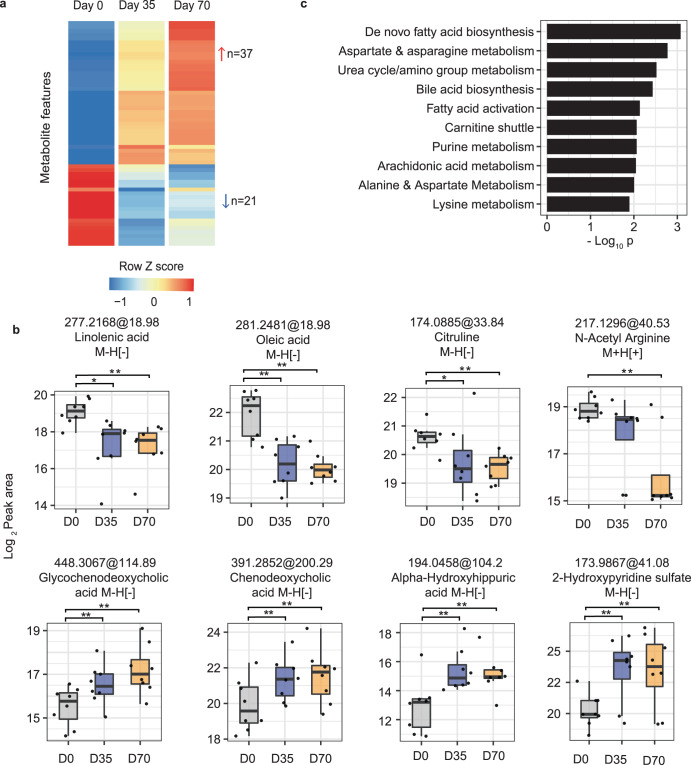
Metabolomic response to metformin in study cohort. **a** Metabolite features significantly different after metformin administration in the plasma samples of participants. Heatmap shows group mean values, for 58 features with FDR < 0.05 and absolute fold change response >1.5 in both post-metformin visits (i.e. day 70 and day 77). **b** Selected significant features, all significant as in (**a**) but also marked by paired *t*-test *p*-values (*p < 0.05, **p < 0.01). The annotation of 2-hydroxypyridine sulfate was based on MS1 and MS2 spectra matches (level 2). The other metabolites were identified with authentic standards (level 1). All of the box plots show the median (center line), first and third quantiles (box limits), and max 1.5 × interquartile range (IQR) from box limits in each direction (upper and lower whiskers). **c** Pathway enrichment of top metabolite features using *mummichog* software (across all modes). Only top ten pathways enriched at *p* < 0.05 and >3 overlapping empirical compounds are shown.

The metabolomic analysis of participants after metformin administration revealed a number of significantly altered features (58 in HILIC ESI+ in Fig. 2a; 179 in HILIC ESI−, 83 in RP ESI+, 176 in RP ESI−, Table 1), with a stringent threshold of false discovery rate (FDR) < 0.05 and fold change >1.5. The group average of the HILIC ESI+ features is shown in Fig. 3a as a heatmap. Among them, 37 metabolite features were increased and 21 decreased consistently in post metformin visits. Examples of individual metabolites from Fig. 3a are shown as box and whisker plots in Fig. 3b. These include urea cycle metabolites citruline and N-acetyl arginine, and bile acids, such as glycochenodeoxycholic acid and chenodeoxycholic acid. Significant pathways impacted by metformin are summarized in Fig. 3c. Several metabolites in de novo fatty acid biosynthesis showed consistent decrease in plasma post metformin treatment (e.g. linolenic acid in Fig. 3b). Decrease of N-acetyl arginine, citrulline and several short-chain and long-chain carnitines (Supplementary Fig. S1) is similar to the observations made by previous metabolomic studies of metformin^33,34^.

### Metabolomic impacts by the seasonal trivalent inactivated influenza vaccine

To analyze the effect of IIV vaccine administration, we focused on the two timepoints before and after vaccination (days 70 and 77 as depicted in Fig. 1), as IIV induces a recall immune response that peaks around one week after vaccination^35^. The day 70 served as vaccination baseline. This was fitted to a mixed effect statistical model with metformin status as a covariate and considering metformin and vaccine interaction (Model 2, described in Methods). With FDR < 0.05 and fold change >1.5, the numbers of significant features are shown in Table 1 for all four LC-MS methods. Of note, Model 2 identified fewer significant features associated with metformin response, because the data points here are cross-sectional comparison with the placebo group, while Model 1 was able to use three time points before vaccination that were matched to the same individuals.

**Fig. 4:**
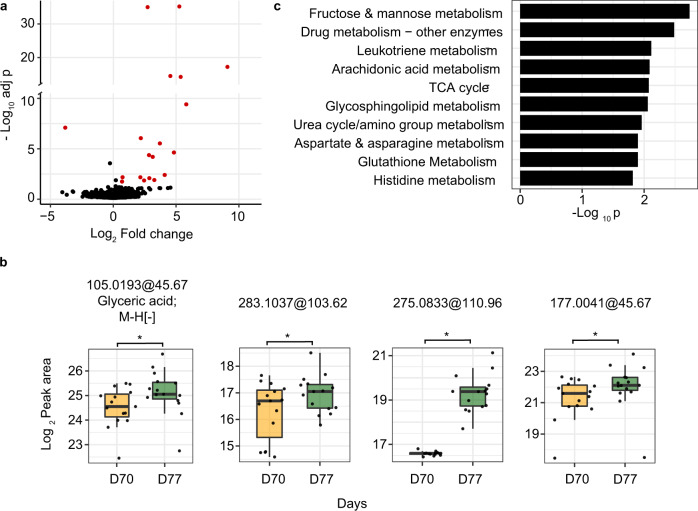
Metabolomic response to seasonal influenza vaccine in study cohort. **a** Metabolite features different after IIV vaccination in the plasma samples of participants, shown in volcano plot with significance on Y-axis and magnitude on X-axis. Significant metabolite features were determined by Model 2 (FDR < 0.05 and absolute fold change response >1.5). **b** Selected significant features, all significant as in (**a**) but also marked by paired *t*-test *p*-values (*p < 0.05, **p < 0.01). Glyceric acid was identified with MSI level 1 annotation and others (1-Methylinosine [283.1037@103.62], Thymidine glycol [275.0833@110.96], Bissulfine [117.0041@45.67]) with MSI level 4 annotation. All of the box plots show the median (center line), first and third quantiles (box limits), and max 1.5 × interquartile range (IQR) from box limits in each direction (upper and lower whiskers). **c** Pathway enrichment of top metabolite features using *mummichog* software (across all modes). Only top ten pathways enriched at *p* < 0.05 and >3 overlapping compounds are shown.

The significant metabolite features associated with vaccine response in the HILIC ESI+ data are shown in Fig. 4a. One of those is glyceric acid (Fig. 4b), a common intermediate of multiple pathways, especially in energy metabolism. It was previously reported to be elevated in autoimmune diseases^36,37^. It’s increase here correlates with the timing of major expansion of antibody secreting cells. Energy metabolism, such as fructose and mannose metabolism and TCA cycle, is indeed enriched in our pathway analysis (Fig. 4c). Pathway analysis also revealed multiple pathways on inflammatory lipid mediators in response to IIV, including leukotriene, arachidonic acids and glycosphingolipids (Fig. 4c). The remaining metabolites in Fig. 4b have a lower-confidence annotation, which may get updated when further information is obtained on these compounds. But they have relatively high abundance in these plasma samples, and their LC-MS peaks are easily verifiable (Supplementary Fig. S2). Therefore, our data indicate that they are true chemical compounds that were elevated after vaccination. Using the same statistical criteria, no significant metabolite was found at days 105 and 140 in comparison to day 70. This was not surprising because most immunological events after IIV occur within the first two weeks^26,38–41^.

### Statistically significant interaction between metformin and IIV was found in metabolomic features

**Fig. 5:**
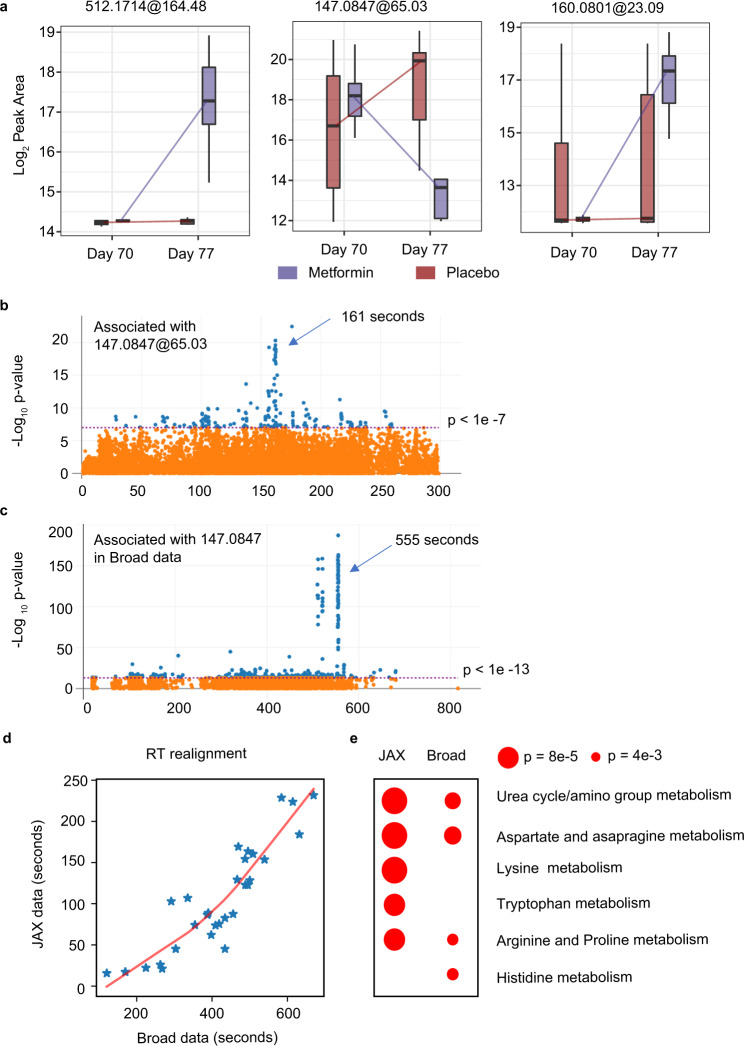
Metabolite features found with significant statistical interaction between metformin and vaccine. **a** Metabolites with FDR < 0.05 in Model 2 show different responses to vaccination based on the metformin treatment. Their m/z@retention time is shown on top. All of the box plots show the median (center line), first and third quantiles (box limits), and max 1.5 × interquartile range (IQR) from box limits in each direction (upper and lower whiskers). **b** Metabolite features associated with [147.0847@65.03] in this study, HILIC ESI+ data. **c** The metabolite features associated with [147.0843@354.85] in the Broad dataset. The *p*-value on Y-axis in (**b**, **c**) is based on Spearman rank correlation. FDR values are in similar range. **d** Retention times between the two studies are comparable after realignment, based on common known metabolites in both datasets. Both are HILIC ESI+ data. **e** Pathway enrichment of metabolites significantly associated with the 147.0843 features in both datasets, as in (**b**) and (**c**). All pathways with *p* < 0.01 are shown.

The above analyses showed that specific metabolic features were impacted by the drug metformin or by the IIV vaccine. To understand if a drug has a positive or negative effect on the vaccination, it is important to know if metabolites are impacted by both. This global metabolomics dataset provides the opportunity to test the statistical interaction between metformin and IIV. This was included as an interaction term in our Model 2. Among four LC-MS methods, 2 in HILIC ESI+ and 1 feature in RP ESI− were found to be significant, using a stringent FDR < 0.05 (Table 1). As shown in Fig. 5, the vaccine responses of these three features are clearly different between the metformin and placebo groups. The m/z values of these features match to a large number of compounds in metabolite databases. The feature 512.1714@164.48 (ESI−) also showed a proper isotopologue pattern. Unfortunately, we failed to identify these three metabolites, but believe they are real compounds because they all passed our filter of background peaks and SNR, and their LC-MS peaks are distinct (Supplementary Fig. S3). Metabolite identification is a common challenge in metabolomics today. Alternatively, functional insight of an unknown metabolite can be gained from metabolome-wide association studies (MWAS)^42,43^.

The MWAS profile of the feature 147.0847@65.03 is shown in Fig. 5b, showing strikingly significant associations to a cluster of compounds that are eluted at 161 s. To validate this in an independent cohort, we retrieved a large dataset of 1172 samples that were analyzed using a similar platform at Broad Institute (Orbitrap mass spectrometer with HILIC ESI+,^44^). Our feature 147.0847@65.03 was matched to 147.0843@354.85 in the Broad study, which has a highly significant association pattern to a cluster of compounds at 556 s (Fig. 5c). Between the two studies, the liquid chromatography had different length, but the retention times were comparable after realignment using known compounds (Fig. 5d). The trendline in Fig. 5d indicates that 65 and 161 s in our study are matched to 354 and 555 s in the Broad study. Therefore, these results indicate that the MWAS pattern of 147.0847@65.03 in our study is reproduced in the Broad data. Indeed, the cluster at 161 s in Fig. 5b and the cluster at 555 s in Fig. 5c share at least five same m/z values. Both clusters, however, have few matches in HMDB, suggesting that the compounds are probably part of the exposome; the feature at 147.0847 is likely to be part of their biological response. These two clusters do not contribute to pathway enrichment tests statistically, because they do not match to known pathways. Yet, the pathway patterns underlying the two overall MWAS results share the same top two pathways (Fig. 5e). Taken together, our feature 147.0847@65.03 showed significant association with urea cycle and aspartate and asparagine metabolism (Fig. 5e); it correlates intriguingly but reproducibly with a group of unknown compounds that warrant future investigation. The other feature 160.0801@23.09 eluted too early to be found in the Broad data (while other major peaks of this m/z match perfectly between two studies).

## Discussion

The metabolic responses to metformin here are consistent with previous reports. The glucose lowering potential of metformin has been largely attributed to its ability to suppress hepatic gluconeogenesis through both AMPK dependent and independent pathways (reviewed in^45^). Interest are also growing in its anti-aging, anti-inflammatory and anti-proliferative roles^46–51^. Metformin reduces pro-inflammatory cytokines and inhibits NF-κB signaling^29,52,53^, both contributing to increased basal inflammation with aging. Overall, metformin regulates several aspects of nutrient sensing and energy homeostasis in various metabolically active organs leading to improved blood glucose and lipid profiles^54,55^. Our data revealed that several lipids and amino acids related pathways were altered by metformin administration. De novo fatty acid biosynthesis was the most significantly altered pathway (Fig. 3b) upon metformin administration in our data. AMPK is one of the key targets of metformin under pharmacological concentrations in liver and have been reported for its role in improved blood glucose and lipid profiles^56,57^. AMPK is also a master regulator of whole-body energy homeostasis and maintains the balance between nutrients supply and energy demand. Liver mediated AMPK phosphorylation of SREBP1 (master regulator of lipogenesis) and ACC1/2 (a rate-limiting enzyme for fatty acid synthesis) inhibits hepatic de-novo lipogenesis^45,58–60^. Moreover, ACC1/2 phosphorylation leads to a decreased production of malonyl-CoA (which is an inhibitor of mitochondrial carnitine palmitoyl transferase 1 (CPT1)) and subsequent enhanced hepatic fatty acid oxidation^45^. Our results showed a decrease of abundance in several metabolites in pathways responsible for synthesis of lipids and their derivatives including de novo fatty acid biosynthesis pathway, arachadonic acid metabolism, glycerophosopholipid metabolism and prostaglandin formation from arachidonate. These results are in line with the previous reports where many lipids and lipid derivatives including poly unsaturated fatty acids (PUFAs), eiconsaids, glycerophospholipds were observed to show decreased plasma abundance upon metformin administration in healthy volunteers^33,34^, and alterations in de-novo fatty acids synthesis and inflammatory lipid derivatives in different pathological conditions^61–66^.

Metformin has been reported to alter the composition of gut microbiota^67–69^, which is expected to change the profile of metabolites of microbial origin. Dahabiyeh et al. showed two microbial metabolites following a similar abundance pattern to metformin administration in healthy volunteers^34^. Our data revealed an increased trend of the plasma metabolites related to bile acids over post metformin visits (Fig. 3c). This is consistent with the report by Hao et al. on an overall increase of plsama bile acids (total, primary, secondary, and unconjugated), along with altered microbiota composition in metformin administered treatment-naïve recently diagnosed diabetic partcipants^67^.

So far, metabolomics has been applied to only few vaccine studies^26,27,39,70^. Thus, the current knowledge of vaccine induced responses was mainly learnt from serological, cellular and transcriptional data^27,38,71,72^. Previous studies of seasonal influenza vaccination in humans revealed common gene signatures of type 1 interferons (between days 1–3) and plasma cells (between days 7–11) corresponding to the induction of innate and adaptive responses, respectively. Many of these studies have highlighted the age dependent differences in vaccine immunogenicity, and our cohort is considered as older adults. Our data revealed several perturbed pathways on carbohydrate and amino acid metabolism 1 week post vaccination. They are in line with previous studies where serine metabolism was shown to be associated with vaccine response^38,39^. Mitochondrial biogenesis and oxidative phosphorylation processes were observed to be impacted by immune responses induced by influenza vaccine in ours and previous reports^40,41^.

In this study, we have focused on day 7 after vaccination. In future opportunities, it will be informative to analyze more time points, including the early ones. The field of immunometabolomics is in its infancy, and it will take time to gain fuller understanding of the vaccine responses in terms of metabolic phenotypes. More and more immunological and vaccine studies take a systems or holistic approach, by collection high throughput multi-omics data, which shall contribute to useful insights on how small molecules, biological or abiological, interact with the immune system. Advantages of metabolomics also include that it’s easy to use biobanked materials, and that it can become very economical in the near future.

We reported the annotation confidence according to the MSI standards^73^. Metabolite identification, however, is still challenging in metabolomics, especially for low-abundance or less common metabolites. This is similar to the early days of genomics, when genes were deposited as unknown sequence but annotation improved over time. The ultrahigh mass resolution of our data also means that people can reuse the data from public repository, and the unknown metabolites, like unknown genes, can gain annotation in the future. This is already demonstrated in our reuse of the Broad dataset^44^: the MWAS of our feature 147.0847@65.03, significant in the interaction between IIV and metformin, is reproduced in the larger study in Fig. 5.

This pilot study was designed to test the interaction between metformin and IIV in a small cohort. Nonetheless, with highly stringent statistical analysis, ultrahigh-resolution metabolomics prove to be powerful to identify (i) metformin and its metabolic signatures in untargeted metabolomics data, (ii) significant metabolic responses to IIV, and (iii) significant metabolites as a result of the interaction between metformin and vaccination. The proof-of-principle is important, demonstrating the feasibility of studying the interaction of drugs and immune responses in human populations.

## Methods

### Clinical study design

This pilot study is a double-blinded placebo-controlled trial in men and women over the age of 65 years. Subjects were screened rigorously for eligibility. Study exclusion criteria included the following: any unstable medical conditions or severe co-morbidities (severe COPD, severe congestive heart failure, advanced neurological disorders, etc), contraindications for metformin (severe renal or liver impairment), contraindication for flu vaccine (history of Guillain-Barre syndrome post vaccination or allergic to component of vaccine), immunosuppressive disorders, immunosuppressive medications, and active cancer. Importantly, participants were excluded if they were prediabetic or diabetic (HbA1c ≥ 5.7%) to avoid any confounding impact of metformin on diabetes status. Eligible participants were randomized to metformin (final dose 1500 mg extended release (ER)/day) or placebo treatment. To limit gastrointestinal issues per current metformin label recommendations, participants started with 1 tablet a day for week 1 (500 mg metformin ER/day or placebo), then 2 tablets a day for week 2 (1000 mg metformin ER or placebo), and finally 3 tablets a day for week 3 until the completion of the study (1500 mg metformin ER or placebo). Fifteen subjects (*n* = 8 metformin, *n* = 7 placebo) were randomized and completed the study on treatment with no differences in basic characteristics at baseline (placebo: 74.71 ± 2.45 years old, 3 males, BMI: 27.31 ± 1.68; metformin: 74.13 ± 2.42 years old, 5 males, BMI: 26.43 ± 1.47).

All participants were vaccinated with Fluzone high-dose trivalent flu vaccine (Sanofi Pasteur Inc., Swiftwater, PA) after ~70 days of treatment. Blood was drawn via standard venipuncture into EDTA-treated vacutainers prior to treatment (Day 0), prior to vaccination (~day 35 and ~day 70), and 7, ~35, and ~70 days post vaccination. The study protocol was approved by the Institutional Review Board at the University of Connecticut Health Center (UCHC) and registered at ClinicalTrials.gov (NCT03996538). All study participants provided written informed consent to participate in the study.

### Plasma sample collection and preparation

EDTA-treated whole blood was immediately centrifuged and the resultant plasma was stored at −80 °C until analyses. Plasma metabolites extraction was carried out by protein precipitation technique using extraction solvent, acetonitrile:methanol (8:1, v/v) containing 0.1% formic acid and isotope labelled [Trimethyl-13C3]-caffeine, [13C5]-L-glutamic acid, [15 N2]-Uracil, [15 N,13C5]-L-methionine, [13C6]-D-glucose and [15N]-L-tyrosine as spike-in controls. 30 μl of plasma was taken and 60 μl of extraction solvent was added. Extraction blanks were also prepared to remove features of non-biological origins. All samples were vortexed and incubated with shaking at 1000 rpm for 10 min at 4 °C followed by centrifugation at 4 °C for 15 min at 20,817 × g. The supernatant was transferred into mass spec vials and 2 μl injected into UHPLC-MS.

### LC-MS metabolomics and LC-MS/MS analysis

The chromatographic separations were performed using Thermo Scientific^TM^ Transcend^TM^ Duo LX-2 UHPLC system interfaced with high resolution Thermo Scientific^TM^ Orbitrap ID-X^TM^ Tribid^TM^ mass spectrometer with a HESI ionization source, using positive and negative ionization modes. All samples were maintained at 4 °C in the autosampler. Data were acquired using hydrophilic interaction liquid chromatography (HILIC) and reversed phase (RP) column in parallel both in positive and negative polarities in full scan mode with mass resolution of 120,000. An Accucore^TM^−150-Amide HILIC column (2.6 μm, 2.1 mm × 50 mm) and a Hypersil GOLD^TM^ RP column (3 μm, 2.1 mm × 50 mm) maintained at 45 °C were used for chromatographic separation. 10 mM ammonium acetate in acetonitrile:water (95:5, v/v) with 0.1% acetic acid as mobile phase A and 10 mM ammonium acetate in acetonitrile:water (50:50, v/v) with 0.1% acetic acid as mobile phase B were used for HILIC method. 0.1% formic acid in water and 0.1% formic acid in acetonitrile were used as mobile phase A and B respectively for RP acquisition. For HILIC acquisition, following gradient was applied at a flow rate of 0.55 ml/min: 0–0.1 min: 0% B, 0.10–5.0 min: 98% B, and 5 min for cleaning and equilibration of column. For RP column, following gradient was applied at a flow rate of 0.4 ml/min: 0–0.1 min: 0% B, 0.10–1.9 min: 60% B, 1.9–5.0 min: 98% B, and 5 min cleaning and column equilibration. This way the mass spec data for each sample was collected consecutively, carrying only one (either HILIC or RP) eluent to the MS for 5 min, while the other eluent was directed to the waste during washing and re-equilibration.

Mass spectrometry data were collected with the following MS settings: mass range, 80–1000 m/z; spray voltage, 3500 V (ESI + ), 2800 V (ESI−); sheath gas, 45 Arb; auxiliary gas, 20 Arb; sweep gas, 1 Arb; ion transfer tube temperature, 325 °C; vaporizer temperature, 325 °C; full scan mass resolution, 120,000 (MS1); normalized AGC target (%), 25; maximum injection time, 100 ms. Data dependent fragmentation (dd-MS/MS) parameters for each polarity as follows: isolation window (m/z), 1.2; stepped HCD collision energy (%), 20,40,80; dd-MS/MS resolution, 30,000; normalized AGC target (%), 20; maximum injection time (ms), 54; microscan, 1; cycle time (sec), 1.2. A full scan data-dependent MS2 (ddMS2) method was utilized to collect MS2 spectra for identification of compounds.

### Metabolomics data processing

All samples were analyzed in a single batch after randomization. Raw LC-MS data was converted to mzML format using ThermoRawFileParser^74^. *Asari* (version 1.9.2), an open source Python software was used for m/z and retention time (rt) alignment, peak detection, feature quantification, and empirical compound based putative identification (level 4 annotation using HMDB)^75^ using the default parameters. Level 1 annotation of compounds were obtained by matching retention times and accurate masses from in-house authentic compound libraries. Level 2 annotation of compounds were obtained by matching acquired MS^2^ spectra of accurate precursor masses from pooled plasma sample against public spectral databases (MassBank, MoNA)^73,76^ using R package Spectra with >=0.7 cosine similarity score. Matching of MS1 features with precursor ion’s accurate masses and retention times was performed using within 10 ppm tolerance and 10 s, respectively. All the metabolite annotation levels adhere to Metabolomics Standard Initiative (MSI) guidelines^73^.

Features were filtered using two criteria. First, the features with three times greater intensity in biological samples than in blanks samples were retained. Second, the features with signal to noise ratio (SNR) greater than 100 were retained (noise in *asari* is defined by the mean of all non-peak data points in an extracted ion chromatogram). Two different quality control samples (a commercial pooled plasma sample and pooled study samples) were used to verify the chromatography and signal reproducibility. Visual inspection of outliers through PCA plots and Total ion count (TIC) was conducted. Data were log2 transformed and mean normalized using top ten percent of high abundance features. Features below detection limit were imputed using half of the minimum intensity value. After QC filtering, 5987, 4245, 3284, 8546 features were retained for downstream data analysis, in each HILIC ESI+, HILIC ESI−, RP ESI+ and RP ESI− modes respectively.

### Statistical analysis

We constructed two linear mixed effect models using the lme4^77^ package in R to assess the metformin response on plasma metabolome, inactivated influenza vaccine response on plasma metabolome and the interaction responses of metformin and vaccine in concert on plasma metabolome.

Model 1: For each metabolite feature, temporal variation due to metformin administration was assessed using linear mixed effects model:

1 $${\rm{Metabolite\; Feature}}\, \sim \,{\rm{day}}+\,(1{\rm{|participant}})$$

The variable ‘day‘ is a categorical variable that indicates discrete time points, including day 0, day 35, and day 70, where day 0 serves as the baseline for metformin administration and day 35 and day 70 represent time points after treatment with metformin. The term (1|participant) controls for repeated measurements on the same participant. The significance of timepoints was assessed with ANOVA and *p*-values were adjusted for multiple-testing based on Storey FDR method^78,79^.

Model 2: In order to quantify the vaccine response and the interaction of metformin and vaccine, we constructed a mixed effect model utilizing a pre-vaccination time point (day 70, vaccination baseline) and a post-vaccination time point (day 77) as follows:

2 $${\rm{Metabolite}}\; {\rm{Feature}} \sim {\rm{day}}+{\rm{metformin}}+\,{{\rm{day}}}^{* }{\rm{metformin}}+\,(1|{\rm{participant}})$$

The ‘day‘ and ‘metformin‘ are used as proxy for vaccine treatment and drug treatment, respectively and modeled as fixed effects. We assessed the differentially abundant metabolite features between pre- (day 70) and post-vaccination (day 77) visits using the *p* value of coefficient term of the variable ‘day‘. The interaction term day*metformin was used to quantify statistically the response as a function of both vaccine and drug administration. The term (1|participant) controls for repeated measurements on the same participant. *P* values assessed using ANOVA were adjusted for multiple-testing based on Storey FDR method^78,79^.

All the above statistical analyses were performed using R version 4.2.0. Pathway enrichment analysis was performed using mummichog (version 2.6.1)^80^ using top metabolite features with *p* value < 0.05.

### Metabolome wide association analysis

Spearman correlation was performed between the features of interest and the remaining features in the same dataset, generating *p*-values for MWAS. FDR was calculated using the Benjamini-Hochberg method. The sample number in this study was 90, in the Broad study 1172. The Broad dataset was retrieved from Metabolomics Workbench (accession number ST001237). The m/z and retention time of identified compounds in the Broad study were obtained from the authors. Known compounds from both studies were compared, then the uniquely matched compounds (same name and m/z within 5 ppm) were used for realigning the retention time.

### Reporting summary

Further information on research design is available in the Nature Research Reporting Summary linked to this article.

## Supplementary information

Supplementary Figures

REPORTING SUMMARY

## Data Availability

The metabolomics data have been submitted to Metabolomics Workbench and publicly available under study id ST002432.
