# Supplementary material for: A pilot metabolomic study of drug interaction with the immune response to seasonal influenza vaccination
Source: NPJ Vaccines. 2023 Jun 12;8:92. doi: 10.1038/s41541-023-00682-2 (PMC10261085; doi:10.1038/s41541-023-00682-2)
Supplement: Supplementary file 1 — Supplementary Figures [file 41541_2023_682_MOESM1_ESM.pdf]

a

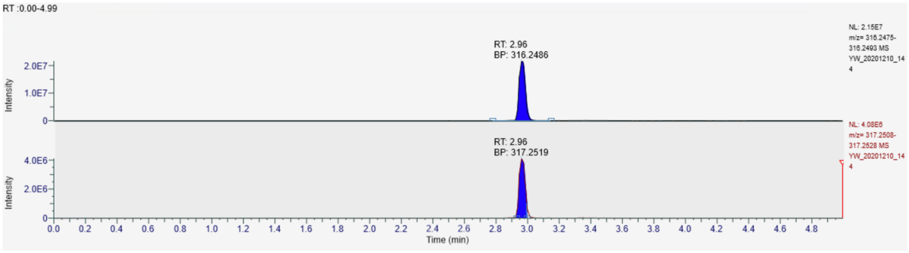

b

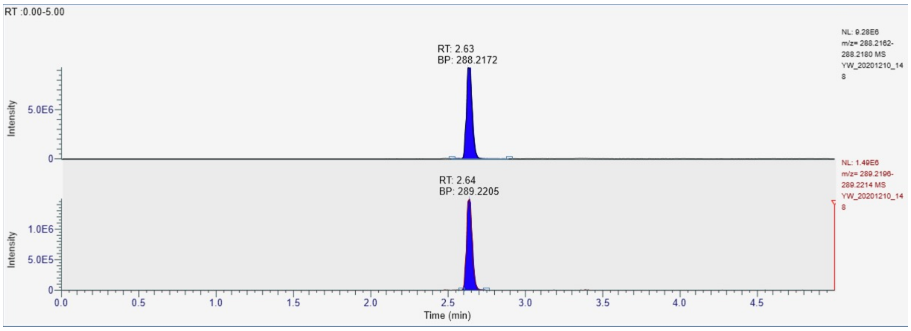

c

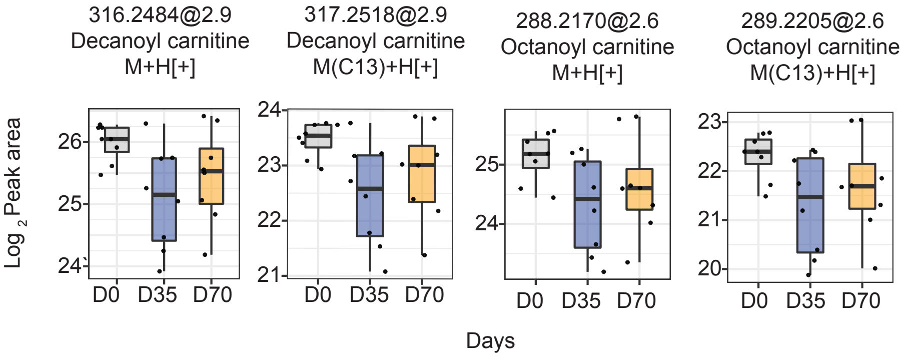

Supplementary Figure S1. Carnitine metabolites in response to metformin administration.

a) Extracted ion chromatograms of ^{12}C and ^{13}C isotopologues of Decanoyl carnitine [317.2518@2.9, as m/z@rt], rt as retention time, here in minutes as from Thermo Scientific software. **b)** Extracted ion chromatograms of ^{12}C and ^{13}C isotopologues of Octanoyl carnitine [289.2205@2.6]. These features (^{13}C isotopologues) were identified with level 2 annotation. **c)** Boxplots of ^{12}C and ^{13}C isotopologues of Octanoyl carnitine and Decanoyl carnitine, showing decreased abundance following metformin administration. All of the box plots show the median (center line), first and third quantiles (box limits), and max 1.5 × interquartile range (IQR) from box limits in each direction (upper and lower whiskers).

a

b

c

d

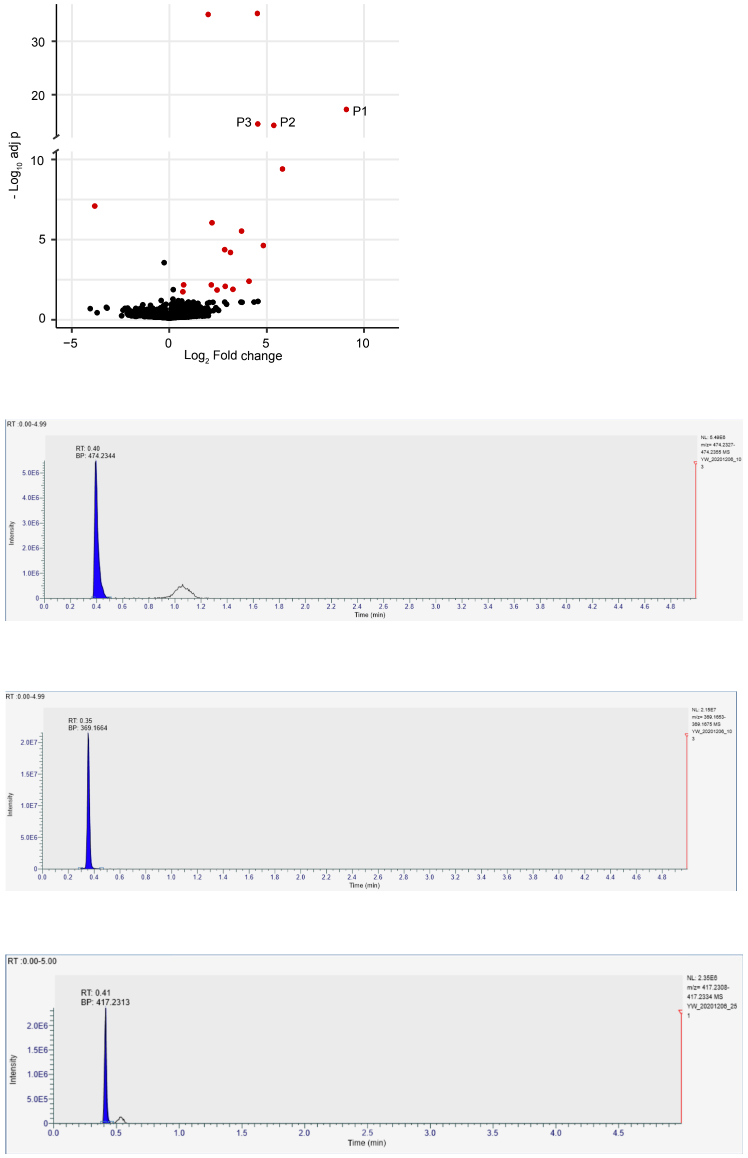

Supplementary Figure S2. Inspection and annotation of top features in Figure 4A.

Extracted ion chromatogram of top significant metabolite features perturbed after IIV vaccination in the plasma samples of participants as determined by **a)** Model 2 (FDR < 0.05 and absolute fold change response > 1.5) are shown in **b)** P1: 474.2341@0.42 **c)** P2: 369.1664@0.35 and **d)** P3: 417.2321@0.4. Retention time in minutes as from Thermo Scientific software.

a

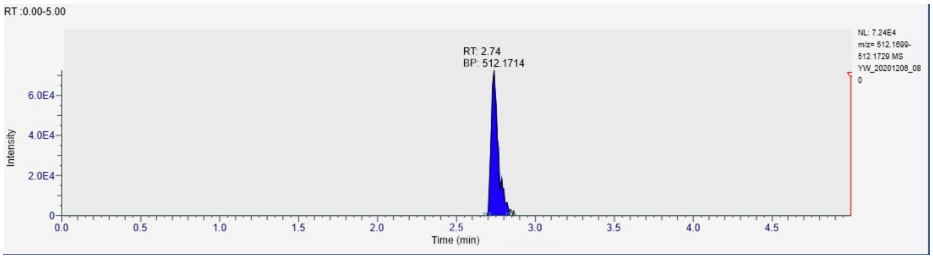

b

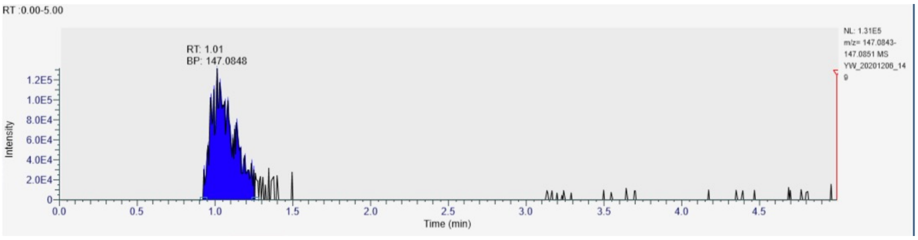

c

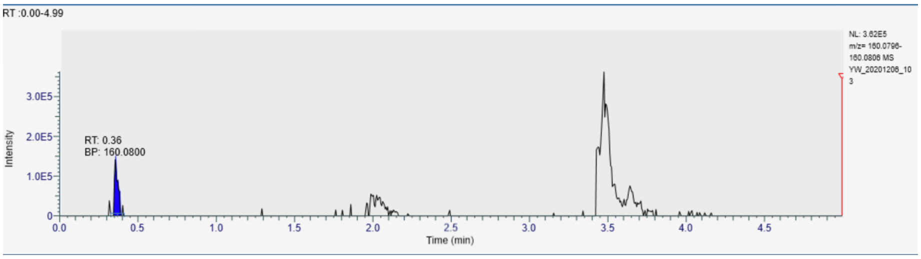

Supplementary Figure S3. Inspection of features in Figure 5.

Extracted ion chromatogram of metabolite features **a)** 512.1714@2.74, **b)** 147.0848@1.01, **c)** 160.0801@0.36) found with significant statistical interaction between metformin and vaccine (FDR < 0.05 in Model 2). Retention time in minutes as from Thermo Scientific software. No smoothing was applied to the figures.
