# Supplementary material for: A pilot metabolomic study of drug interaction with the immune response to seasonal influenza vaccination
Source: NPJ Vaccines. 2023 Jun 12;8:92. doi: 10.1038/s41541-023-00682-2 (PMC10261085; doi:10.1038/s41541-023-00682-2)
Supplement: Supplementary file 2 — REPORTING SUMMARY [file 41541_2023_682_MOESM2_ESM.pdf]

nature portfolio

Corresponding author(s): Shuzhao Li

Last updated by author(s): Apr 29, 2023

Reporting Summary

Nature Portfolio wishes to improve the reproducibility of the work that we publish. This form provides structure for consistency and transparency in reporting. For further information on Nature Portfolio policies, see our Editorial Policies and the Editorial Policy Checklist.

Statistics

For all statistical analyses, confirm that the following items are present in the figure legend, table legend, main text, or Methods section.

n/a	Confirmed	
☐	☒	The exact sample size (*n*) for each experimental group/condition, given as a discrete number and unit of measurement
☒	☐	A statement on whether measurements were taken from distinct samples or whether the same sample was measured repeatedly
☐	☒	The statistical test(s) used AND whether they are one- or two-sided<br>*Only common tests should be described solely by name; describe more complex techniques in the Methods section.*
☐	☒	A description of all covariates tested
☒	☐	A description of any assumptions or corrections, such as tests of normality and adjustment for multiple comparisons
☒	☐	A full description of the statistical parameters including central tendency (e.g. means) or other basic estimates (e.g. regression coefficient) AND variation (e.g. standard deviation) or associated estimates of uncertainty (e.g. confidence intervals)
☒	☐	For null hypothesis testing, the test statistic (e.g. *F*, *t*, *r*) with confidence intervals, effect sizes, degrees of freedom and *P* value noted<br>*Give P values as exact values whenever suitable.*
☒	☐	For Bayesian analysis, information on the choice of priors and Markov chain Monte Carlo settings
☒	☐	For hierarchical and complex designs, identification of the appropriate level for tests and full reporting of outcomes
☒	☐	Estimates of effect sizes (e.g. Cohen's *d*, Pearson's *r*), indicating how they were calculated

Our web collection on statistics for biologists contains articles on many of the points above.

Software and code

Policy information about availability of computer code

Data collection	Thermo Scientific Xcalibur 4.3
Data analysis	R 4.2.0

For manuscripts utilizing custom algorithms or software that are central to the research but not yet described in published literature, software must be made available to editors and reviewers. We strongly encourage code deposition in a community repository (e.g. GitHub). See the Nature Portfolio guidelines for submitting code & software for further information.

Data

Policy information about availability of data

All manuscripts must include a data availability statement. This statement should provide the following information, where applicable:

- Accession codes, unique identifiers, or web links for publicly available datasets
- A description of any restrictions on data availability
- For clinical datasets or third party data, please ensure that the statement adheres to our policy

The metabolomics data have been submitted to Metabolomics Workbench, and will be made public at the time of publication.

Research involving human participants, their data, or biological material

Policy information about studies with human participants or human data. See also policy information about sex, gender (identity/presentation), and sexual orientation and race, ethnicity and racism.

Reporting on sex and gender	8 males, 7 females. No sex or gender based analysis. Small pilot study.
Reporting on race, ethnicity, or other socially relevant groupings	NA. Small pilot study.
Population characteristics	Age distribution provided. No other information was relevant. Small pilot study.
Recruitment	This pilot study is a double-blinded placebo-controlled trial in men and women over the age of 65 years. Subjects were screened rigorously for eligibility. Study exclusion criteria included the following: any unstable medical conditions or severe co-morbidities (severe COPD, severe congestive heart failure, advanced neurological disorders, etc), contraindications for metformin (severe renal or liver impairment), contraindication for flu vaccine (history of Guillain-Barre syndrome post vaccination or allergic to component of vaccine), immunosuppressive disorders, immunosuppressive medications, and active cancer. Importantly, participants were excluded if they were prediabetic or diabetic (HbA1c ≥ 5.7%) to avoid any confounding impact of metformin on diabetes status.
Ethics oversight	The study protocol was approved by the Institutional Review Board at the University of Connecticut Health Center (UCHC) and registered at ClinicalTrials.gov (NCT03996538).

Note that full information on the approval of the study protocol must also be provided in the manuscript.

Field-specific reporting

Please select the one below that is the best fit for your research. If you are not sure, read the appropriate sections before making your selection.

☒ Life sciences ☐ Behavioural & social sciences ☐ Ecological, evolutionary & environmental sciences

For a reference copy of the document with all sections, see nature.com/documents/nr-reporting-summary-flat.pdf

Life sciences study design

All studies must disclose on these points even when the disclosure is negative.

Sample size	Pilot study. Fifteen subjects (n=8 metformin, n=7 placebo) were randomized and completed the study on treatment with no differences in basic characteristics at baseline.
Data exclusions	None.
Replication	NA.
Randomization	Fifteen subjects (n=8 metformin, n=7 placebo) were randomized and completed the study on treatment with no differences in basic characteristics at baseline
Blinding	This pilot study is a double-blinded placebo-controlled trial in men and women over the age of 65 years.

Reporting for specific materials, systems and methods

We require information from authors about some types of materials, experimental systems and methods used in many studies. Here, indicate whether each material, system or method listed is relevant to your study. If you are not sure if a list item applies to your research, read the appropriate section before selecting a response.

Materials & experimental systems

n/a	Involved in the study	
☒	☐	Antibodies
☒	☐	Eukaryotic cell lines
☒	☐	Palaeontology and archaeology
☒	☐	Animals and other organisms
☒	☐	Clinical data
☒	☐	Dual use research of concern
☒	☐	Plants

Methods

n/a	Involved in the study	
☒	☐	ChIP-seq
☒	☐	Flow cytometry
☒	☐	MRI-based neuroimaging
